# Systemic glucose levels are modulated by specific wavelengths in the solar light spectrum that shift mitochondrial metabolism

**DOI:** 10.1371/journal.pone.0276937

**Published:** 2022-11-03

**Authors:** Michael B. Powner, Glen Jeffery

**Affiliations:** 1 Centre for Applied Vision Research, School of Health & Psychological Sciences, City, University of London, London, United Kingdom; 2 UCL Institute of Ophthalmology, University College London, London, United Kingdom; Massachusetts General Hospital, UNITED STATES

## Abstract

Systemic glucose levels can be modulated with specific solar wavelengths that influence mitochondrial metabolism. Mitochondrial respiration can be modulated using light that shifts ATP production with exceptional conservation of effect across species, from insects to humans. Known wavelengths have opposing effects of photobiomodulation, with longer wavelengths (660–900 nm red/infrared) increasing ATP production, and 420 nm (blue) light suppressing metabolism. Increasing mitochondrial respiration should result in a greater demand for glucose, and a decrease should result in a reduced demand for glucose. Here we have tested the hypothesis that these wavelengths alter circulating glucose concentration. We first established an oral glucose tolerance test curve in a bumblebee model, which showed sustained increase in systemic glucose beyond that seen in mammals, with a gradual normalisation over eight hours. This extended period of increased systemic glucose provided a stable model for glucose manipulation. Bees were starved overnight and given a glucose load in the morning. In the first group glucose levels were examined at hourly intervals. In the second group, bees were additionally exposed to either 670 nm or 420 nm light and their blood glucose examined. Increasing mitochondrial activity with 670 nm light at the peak of circulating glucose, resulted in a significant 50% reduction in concentration measured. Exposure to 420nm light that retards mitochondrial respiration elevated systemic glucose levels by over 50%. The impact of 670 nm and 420 nm on mitochondria is highly conserved. Hence, different wavelengths of visible light may be used to modulate systemic metabolism bidirectionally and may prove an effective agent in mammals.

## Introduction

Optical stimulation of mitochondria with specific wavelengths present in natural light modulates their respiration rate, ATP production and impacts on general physiology, a process known as photobiomodulation. 420 nm light is absorbed by mitochondria [1], specifically the chromophore porphyrin located in the inner mitochondrial membrane. Following 420 nm absorption, porphyrins interact with molecular triplet oxygen to form singlet oxygen, superoxide anion, and hydroxyl radicals. These reactive oxygen species reduce mitochondrial respiration rate, disrupt function, and can lead to cell death [2]. 670 nm and other longer wavelengths differs in their impact. They are not absorbed by mitochondria directly [1, 3, 4]. Instead, the mechanism of action is proposed to be via light absorption by nanoscopic interfacial water layer surrounding mitochondrial ATP rotor pumps. This reduces the waters viscosity, allowing the rotor pump to achieve greater momentum. In support of this, there is marked overlap between the spectrum of water absorption of longer wavelengths and improved mitochondrial function [5]. The effect on mitochondria from red and blue light appears universal across species, including insects and humans [Red-light:^6^–^9^], [Blue-light:^1^, ^10^, ^11^].

These two visible light interventions have different effect time courses reflecting their different mechanisms of action. Effective 670 nm light exposures require only short duration, down to one minute. With a single exposure eliciting improved function for up to five days [8, 12]. Repeated 420 nm light exposure for 15 minutes impact’s mitochondrial function, decreasing it over a couple of days if exposed daily. However, there is a rebound effect [1]. This rebound is proposed to be a response of mitochondrial respiratory complexes to compensate for repeated, restricted insult, by boosting activity. Hence, extended 420 nm light exposures are required to maintain a suppression of mitochondrial activity.

Mitochondrial ATP production requires a supply of substrate, the primary source in animals being glucose, released into the blood from stores, or immediate uptake through digestion. Hence, an increase in mitochondrial activity should be associated with will lower systemic glucose level. While the converse should be associated with 420nm exposure.

Here we report that visible light modulates systemic glucose levels in bumblebee haemolymph.

## Methods

### Animals

Bumblebees (Bombus *terrestris*) colonies were obtained from Koppert UK. Each experimental group contained replicates from two colonies. The same ratio of bees from each colony were used per experiment. Bumblebee colonies were maintained *ad libitum* on 50% sucrose solution in water, and pollen, at a constant room temperature of 21°C.

### Oral glucose tolerance test

Bumblebees were transferred to plastic containers (190x143x120 mm) and kept in the dark. Those from different colonies were not mixed, and ~10 bees were housed in a box per experiment. They were fasted for 14 hours overnight, with access to water *ad libitum*. An adapted oral glucose tolerance test (OGTT) commenced at 9am the following morning; individual bumblebees were then housed separately in 50 ml tubes and were given 70 μl 50%(w/v) glucose water. Bees that did not consume all 70 μl within seven minutes were discarded. Water was provided *ad libitum* post glucose challenge. Haemolymph glucose concentration was measured periodically following glucose challenge. Haemolymph was collected; an antenna was detached and haemolymph outflow induced by pressing the abdomen, resulting in a bead forming at the antenna base [8, 13]. A blood glucose monitor (Kinetik Wellbeing, UK) was used to measure glucose concentration, as verified in bees [14]. Two measurements were averaged (intra-measurement variance: 1.1mmol/L). To establish an OGTT response curve, timepoints of 0, 0.5, 1, 2, 3-, 4-, 6- and 8-hours post glucose challenge were assessed, with ≥18 bees for each data set. Two animals, two hours post glucose challenge, measured glucose levels above the range of the glucose monitor, and one animal similarly at the three-hour timepoint. Data from these were discarded.

### Solar light exposure

**Fig 1:**
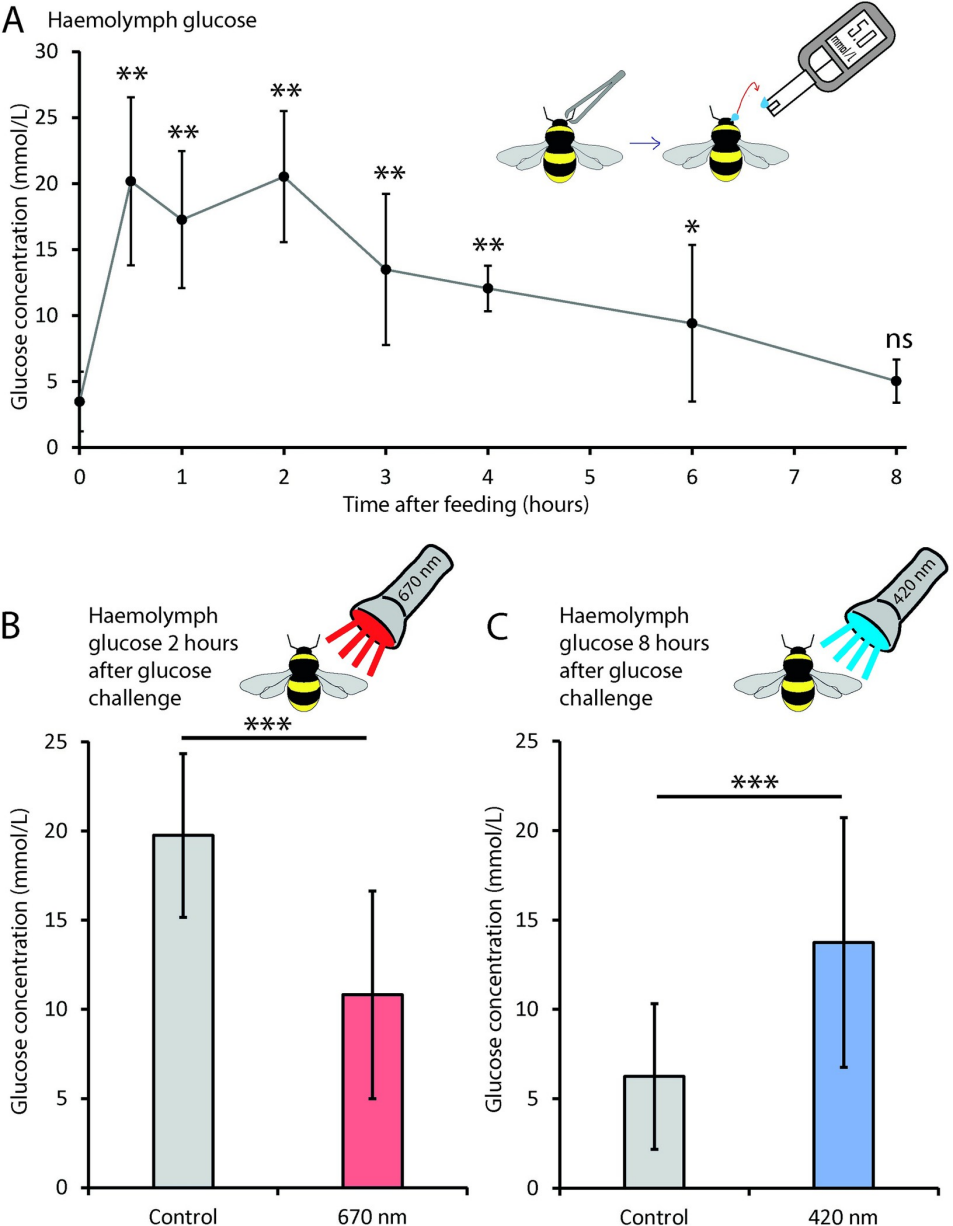
Visible light controls bumblebee haemolymph glucose levels. An oral glucose tolerance test (OGTT) response was established (A), showing sustained elevated systemic glucose levels (significant difference at timepoints ≤6 hours compared to 0 hours). Thus, providing an extended timeframe model, compared to a mammalian OGTT response, to assess optical modulation these systemic sugars. Exposure to 670 nm light (B) significantly suppressed the peak glucose concentration seen systemically at two hours post glucose challenge (*p = 0*.*0008*), compared to control bees. The opposite effect was observed with 420 nm light (C); systemic haeomlymph glucose levels did not return to starvation levels eight hours post glucose challenge, remaining significantly different from controls (*p< 0*.*0001*). Visible light influences glucose concentration in the systemic circulation. Red light reduces peak systemic loading, while blue can extend systemic exposure to high glucose concentrations. Haemolymph glucose concentration was assessed using a commercial blood glucose meter (schematic in A). *; p< 0.05, *; p< 0.01, ***; p< 0.001, ns; not significant. Error bars are standard deviations.

Peak ATP upregulation in response to 670 nm exposure in invertebrates is three hours post exposure [9]. To assess the effect of 670 nm light on peak glucose concentration during the OGTT, bumblebees (≥20 animals per group) were exposed to 670 nm light (40mW/cm^2^) for 15 minutes (36 J/cm^2^), three hours before the timepoint of peak glucose concentration during OGTT. The OGTT curve established here (Fig 1A), peaks two hours post glucose challenge, as such 670 nm exposure started one hour prior to glucose challenge. Two animals measured glucose levels above the range of the monitor in the control group and were discarded. 420 nm light exposure (40mW/cm^2^) was delivered throughout the duration of the OGTT (eight hours, 1152 J/cm^2^, ≥25 bumblebees per group) because mitochondrial respiratory complexes can rebound rapidly following restricted insults [1]. One animal in this control group measured below the limit of the glucose monitor, and one animal recorded above the limit of detection by the monitor in the 420 nm group and were discarded. In both cases light was delivered via LEDs that had a half power band of approximately 10nm. Hence, 670 nm and 420 nm were the peak wavelengths within a range of light exposure. Bumblebees were housed individually in 50 ml clear plastic tubes during both light exposure regimes, with LEDs illuminating the dorsal aspect of the bee from a distance of 120mm. Light exposure does not induce a change of air temperature within the tubes.

### Statistical analysis

Kruskal-Wallis H test and post-hoc Dunn’s multiple comparison tests assessed significance between groups. Bonferroni correction for multiple comparisons was applied. Error bars are standard deviations.

## Results

### Oral glucose tolerance test curve

Bumblebees respond to a fasting glucose challenge with the expected rise in haemolymph glucose concentration, peaking at two hours (Fig 1A). Systemic glucose levels slowly decrease over the next six hours but remained significantly elevated compared to fasting levels. These only returned to fasting glucose levels eight hours post challenge (Fig 1A). Hence, bumblebees maintain high systemic glucose levels for extended durations after glucose loading, making them a suitable candidate for investigating optical interventions.

### Effect of solar light on systemic glucose

Having established a glucose tolerance test timeline for the bumblebee, we established the effect that optical interference of mitochondrial activity has on glucose levels with long and short wavelengths.

670 nm light exposure significantly reduced the peak haemolymph glucose concentration by 45%, two hours after glucose challenge (Fig 1B, *p = 0*.*0008*). In contrast, 420 nm light, known to inhibit mitochondrial respiration, reduced glucose clearance from haemolymph. Here there was an average 54.5% increase in glucose levels, eight hours post glucose challenge in 420 nm light exposed bumblebees compared to controls (Fig 1C, *p< 0*.*0001*).

## Discussion

Here, we report that selective wavelengths of solar light modulate systemic glucose concentration in a bidirectional manner. 670 nm red light reduces peak glucose concentrations after feeding, while 420 nm blue light prolongs the duration of elevated glucose levels post feeding.

Red light photobiomodulation has many beneficial effects, including increased insect mobility with age [7, 15] and extension of insect lifespan [7, 15]. The extension of bumblebee lifespan after 670 nm light and mobility, we observed previously [7], may be linked to the tighter regulation of glucose, as displayed here, however it would be naïve to assume that red light only shifts ATP production and does not influence other intra or extra cellular signaling critical for function, including general metabolism [16, 17].

Changing ATP production requires substrate, the primary of which for bees is sugar circulating in the haemolymph, as the bee has limited glycogen stores in flight muscles or fat body [18]. Upon feeding, glucose is transported from the crop to the haemolymph and if glucose levels exceed demand, is converted into trehalose by the fat body for storage. Trehalose is broken down into glucose, depleting reserves when demand increases during starvation [19]. A dynamic response of glucose also occurs during protracted periods of feeding or fasting [20]. Like mammals, insects possess peptide hormones that regulate these circulating carbohydrates, and the storage of lipid and glycogen in fat body and muscle. One example of which is a functional homolog of glucagon; adipokinetic hormone, stimulates trehalose release from glycogen, and diacylglycerol from triacylglycerol within the fat body [19]. Light modulation of glucose levels most likely represents a change in cell uptake and oxidation rate of it by mitochondria here, given the known mechanism; and supported by previous reports of increased respiration rate in healthy bumblebees following 670 nm light exposure [12]. However, an effect on trehalose metabolism is possible.

In contrast to 670 nm light exposure in bumblebees, increasing drosophila haemolymph glucose levels by disrupting insulin-like ligand regulation is correlated with increasing lifespan of the animal [21]. However, other physiological responses occur. For example, the model used by Broughton et al. is also reported to have lower fertility rates, a phenotype correlated with increased lifespan of flies [21]. 420 nm blue light decreases lifespan and induces neurodegeneration in drosophila [22] and reduces mobility [1]. Even though blue light increases haemolymph glucose levels, blue light exposure also generates reactive oxygen species and lipid peroxidation in drosophila [23], and stress-response genes in photoreceptor cells of the fly retina [24]. It is unlikely that increased haemolymph glucose levels induced by solar light would have similar effects to that shown by altering hormonal control of glucose metabolism. Long-term exposure to 420 nm light would likely reduce bee lifespan and mobility, and exposure to 670 nm light increases lifespan and mobility [7].

Given the increased expenditure of glucose in flight [25], bumblebees were restricted in movement during OGTT. Thus, glucose challenge responses represent those at rest. The large capacity for sugar concentration within bee homeostasis [26], and the rest state of the bees fit with the long duration of elevated glucose levels measured (Fig 1A). The two-hour delay until peak glucose level represents the initial storage capacity of the crop with gradual release into the haemolymph [20].

Here we irradiate the whole body of the insect, which may be unrealistic in field conditions, or translation to other species. However, 670 nm exposure to restricted regions of the body has been shown to induce an abscopal effect [27]. Light must also penetrate the exoskeleton in invertebrates prior to cellular/mitochondrial stimulation, and yet consistent effective exposure times and intensity are observed across species for both wavelengths of light [1, 8, 12]. The mechanism of action is conserved across species [human, 5, 28; mouse, 29–31; drosophila, 15; non-mammalian review, 32]. As such, results from insects are likely translatable in principle through to human. The abscopal effect, along with the added recent discovery that humans have circulating, cell-free floating mitochondria [33, 34] in blood, provides a route for whole body stimulation from localised transcutaneous stimulation. As such, bidirectional control of systemic glucose levels might be achievable using visible light, offering a non-contact, non-pharmaceutical option, which warrants further investigation.
